# Xiao *et al*.: first *in-situ* temperature measurements at the far side of the lunar surface

**DOI:** 10.1093/nsr/nwac185

**Published:** 2022-09-08

**Authors:** Norbert I Kömle

**Affiliations:** Space Research Institute, Austrian Academy of Sciences, Austria

Knowledge of the thermal properties of material forming the near-surface layers of planetary bodies is of utmost importance both from a scientific and a technical point of view. On the Moon, the surface is covered by a several-metres-thick layer of so-called *regolith*, a fine powder made up mainly of irregular mineral particles in the micrometre size range. Such powders are highly porous and the grain size distribution as well as the geometric shape of the particles controls their thermal properties. In particular, under vacuum conditions, as on the lunar surface, such a regolith layer exhibits an extremely small thermal conductivity, of the order of 10^−4^–10^−3^ W/m/K, and thus is a very good thermal isolator.

The research article by Xiao *et al*. [1] presents the first analysis of the temperature measurements at the far side of the lunar surface, conducted as part of the Chang’E-4 mission. The authors have carefully evaluated the thermal conductivity of the far-side lunar regolith at the landing site of the Chang’E-4 spacecraft from the data available to them at the time.

Looking forward, however, an extension of this investigation over a longer time would be highly desirable, in order to confirm their results, since the present investigation includes only one month of data, while—as far as I know—Chang’E-4 has provided temperature data over a much longer period. Moreover, an extension of the thermal model from a simple 1D analysis to a full 3D model using finite element solvers like COMSOL or ANSYS would also be an interesting continuation of the present work. The applied methods have the potential to perform similar analyses in future samples—maybe from the polar areas, where several space agencies plan to build a manned lunar base in the near future. For such a project, the thermal properties of the surface regolith provide very relevant data. As an example, we mention a recent design study for a lunar habitat by Herzig *et al*. (2022), described in reference [2] (also at https://www.youtube.com/watch?v=1rOLgS_StSc&t=234s). It consists of toroidal-shaped units connected by a tunnel system. Here, the whole habitable area (greenhouses, tunnels and living rooms) is covered by several metres of the local surface regolith, for protection from harmful cosmic radiation and to avoid too much cooling during dark periods. Figure 1 shows a surface view of the suggested habitat.

**Figure 1.**
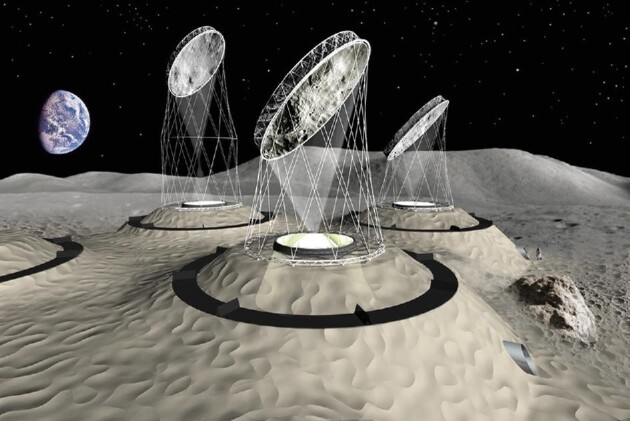
Example of a lunar base consisting of inflatable elements, which are protected by several meters of natural lunar regolith (source: Herzig *et al*. [2]).

***Conflict of interest statement*.** None declared.

## Author Biographical

(Retired from) Space Research Institute, Austrian Academy of Sciences, Austria.
